# Development and validation of a device for monitoring laryngeal motion during swallowing

**DOI:** 10.3389/frobt.2023.1259257

**Published:** 2023-11-02

**Authors:** Keiko Aihara, Yoko Inamoto, Eiichi Saitoh, Seiko Shibata, Yuriko Sato, Maki Harada, Yohei Otaka

**Affiliations:** ^1^ Faculty of Rehabilitation, School of Health Sciences, Fujita Health University, Toyoake, Japan; ^2^ Department of Rehabilitation Medicine I, School of Medicine, Fujita Health University, Toyoake, Japan; ^3^ Department of Rehabilitation, Fujita Health University Hospital, Toyoake, Japan

**Keywords:** hyoid, larynx, swallowing, deglutition, feedback, rehabilitation

## Abstract

**Objectives:** Hyolaryngeal movement during swallowing is essential to airway protection and bolus clearance. Although palpation is widely used to evaluate hyolaryngeal motion, insufficient accuracy has been reported. The Bando Stretchable Strain Sensor for Swallowing (B4S™) was developed to capture hyolaryngeal elevation and display it as waveforms. This study compared laryngeal movement time detected by the B4S™ with laryngeal movement time measured by videofluoroscopy (VF).

**Methods:** Participants were 20 patients without swallowing difficulty (10 men, 10 women; age 30.6 ± 7.1 years). The B4S™ was attached to the anterior neck and two saliva swallows were measured on VF images to determine the relative and absolute reliability of laryngeal elevation time measured on VF and that measured by the B4S™.

**Results:** The intra-class correlation coefficient of the VF and B4S™ times was very high [ICC (1.1) = 0.980]. A Bland–Altman plot showed a strong positive correlation with a 95% confidence interval of 0.00–3.01 for the mean VF time and mean B4S™ time, with a fixed error detected in the positive direction but with no proportional error detected. Thus, the VF and B4S™ time measurements showed high consistency.

**Conclusion:** The strong relative and absolute reliability suggest that the B4S™ can accurately detect the duration of superior-inferior laryngeal motion during swallowing. Further study is needed to develop a method for measuring the distance of laryngeal elevation. It is also necessary to investigate the usefulness of this device for evaluation and treatment in clinical settings.

## Introduction

Hyolaryngeal movement during swallowing is essential to airway protection and bolus clearance. Decreased hyolaryngeal elevation leads to incomplete airway closure and reduced opening of the upper esophageal sphincter, which may result in aspiration, penetration, and/or pharyngeal residue (Steele et al., 2011). Therefore, evaluating hyolaryngeal movements during swallowing is critical not only for detecting the presence or absence of dysphasia but also for understanding its underlying pathophysiology. Hyolaryngeal measurement is important because hyolaryngeal movement is the only motion during swallowing that is palpable, so it can be used as training feedback as well as an outcome measure of therapeutic procedures.

In the clinical setting, the four-finger method is widely used to palpate the hyoid and evaluate laryngeal movement (Logemann, 1998). It enables oral transit time and delayed pharyngeal response to be roughly estimated from tongue dynamics and hyolaryngeal movement (Logemann, 1998). Although palpation can be simply and easily performed in the clinic, questions remain about its reliability and accuracy. In a report investigating the intra- and inter-rater reliability of hyolaryngeal palpation during swallowing, intra-rater reliability was substantial for thin liquids but low for thick liquids, and inter-rater reliability was reported to be insufficient (McCullough et al., 2000). Imaging evaluation such as videofluoroscopy (VF) and swallowing computed tomography, on the other hand, enables to visualize the hyolaryngeal movement and to quantify its motion. There are, however, disadvantages of invasiveness such as radiation exposure and barium ingestion, and of time and location limitations. Therefore, there is a need to develop evaluation methods that are quantitative, reliable, and highly usable in daily clinical practice.

The ability to perform reliable objective assessments is also a major advantage to clinicians during intervention. Hyolaryngeal movement, as the only externally detectable movement during swallowing, is also regarded as important in the point that providing feedback to both dysphagia patients and clinicians. Attempts have been made to capture hyolaryngeal movements with surface electromyography (sEMG) or reflective photosensors for feedback (Bogaardt et al., 2009; Furuta et al., 2012; Sato et al., 2012; Athukorala et al., 2014; Azola et al., 2015; Rangarathnam and McCullough, 2016; Azola et al., 2017). These techniques have often been used to help patients acquire a swallow in which they can voluntarily adjust the range and/or duration of hyoid and larynx movement (Huckabee and Cannito, 1999; Carnaby-Mann et al., 2010; McCullough et al., 2012; McCullough and Kim, 2013). However, sEMG is not easy to prepare, nor is it easy to interpret hyolaryngeal movements directly from the displayed muscle activity waveforms, and thus the use of sEMG for real-time biofeedback during training is not always available to train swallowing maneuvers. A simple and objective method for evaluating the superior hyolaryngeal movement, along with a method for visualizing this movement, might be effective both for providing feedback and performing quantitative evaluation of swallowing. Although a method using reflective photosensors may be easy for patients to understand because it captures actual laryngeal movements, the equipment is large and not easily portable and does accurately capture the movement of the larynx in patients with a thick layer of subcutaneous fat in the anterior neck or in women with small laryngeal ridges. A recently proposed laryngeal motion detection device using a Piezo pressure sensor (Ono et al., 2010; Iizuka et al., 2018) has been reported to be able to evaluate the duration of laryngeal elevation almost accurately even in subjects with no apparent laryngeal prominence. However, to the best of our knowledge, it is not yet ready for clinical use.

We believe that a device that is not only simple to operate but also reliable and capable of objective assessment in clinical setting would overcome the disadvantages of conventional qualitative assessment of hyolaryngeal movement by palpation and feedback by time-consuming methods that require discipline to master. To achieve this, we have developed the new Bando Stretchable Strain Sensor for Swallowing (B4S^™^), which can easily capture superior hyolaryngeal movement and can be used in the clinical setting.

## Materials and methods

The study was conducted at a university hospital in Japan and it compared the concomitant validity of measurements taken by the newly developed B4S^™^ with the those taken by VF. The study protocol was approved by the institutional review board of our university (CR-18-002) and was conducted in accordance with the principles of the Declaration of Helsinki of 1964, as revised in 2013, and ethical guidelines for medical and health research involving human subjects.

Participants were 20 healthy young adults without any swallowing difficulties (10 men, 10 women; mean ± standard deviation age, 30.6 ± 7.1 years). Exclusion criteria were age <20 years and history of stroke, neuromuscular disease, respiratory disease, or cancer. All participants provided written informed consent prior to participation in the study.

**FIGURE 1:**
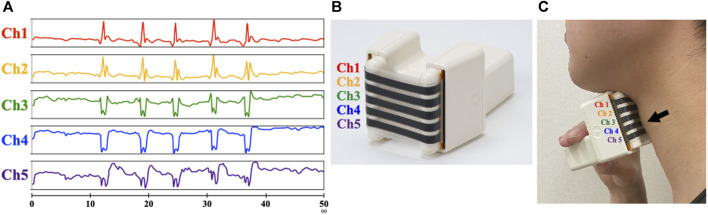
Design of the BANDO Stretchable Stain Sensor for Swallowing (B4S™). **(A)** Display screen on the Tablet **(B)** Device with five C-STRECH^®^ sensors arranged vertically. **(C)** Attachment of device during measurement Arrow in the figure indicate laryngeal prominence.

**FIGURE 2:**
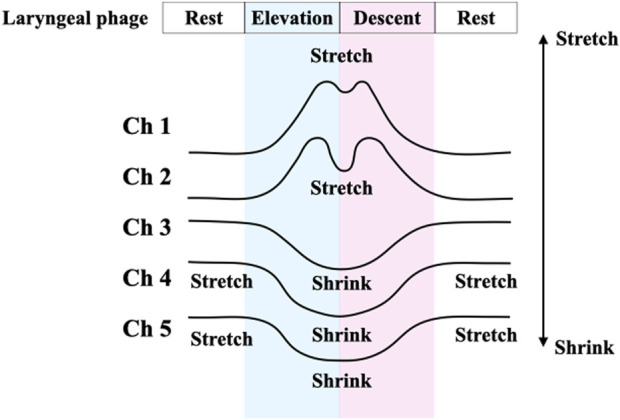
Schematic diagram of the swallowing waveform measured by B4S™. Schematic diagram of the swallowing waveform measured with B4S™. The upward waveform is displayed when the sensor is stretched, and the downward waveform is displayed when the stretched state returns to the neutral state.

The B4S^™^ system comprises a device and a tablet computer. The device has five C-STRETCH^®^ sensors (Nakamoto et al., 2015) aligned vertically (Ch1–Ch5, from top to bottom). The highly resilient strain gauge sensor detects the magnitude of elongation. The B4S^™^ was used to measure vertical movements of the hyoid and larynx, which can be evaluated more consistently than in other directions on palpation. When the device is worn on the front of the neck during swallowing, the sensors stretch or contract, thereby sensing the laryngeal elevation and decent that is visibly apparent. The stretching and contraction of each sensor is displayed in real time as five waveforms in a dedicated tablet app (Figure 1). To monitor swallowing, the Ch4 sensor is aligned over the laryngeal ridge prior to measurement. During swallowing, the laryngeal ridge passes sequentially from Ch4 to Ch3, Ch2, and Ch1 as the larynx elevates and it passes again over Ch1, Ch2, Ch3, and Ch4 as the larynx descends. Each sensor detects the elongation and contraction caused by the laryngeal elevation and descent, which is indicated as a waveform and is used to identify swallowing (Figure 2).

Each participant performed two saliva swallows (SS) while wearing the B4S^™^ on the anterior surface of the neck under VF. The position of the B4S^™^ on the neck was adjusted by the examiner so that the Ch4 sensor was positioned on the laryngeal ridge at rest. Then, each participant performed two SS with a 30-s interval between them. An X-ray fluoroscope (PVW-30A; Toshiba, Tokyo, Japan), digital video recorder (WV-D900; Sony, Tokyo, Japan), and video monitor (OEV-143; Olympus, Tokyo, Japan) were used. A lead ball with a diameter of 10.5 mm was attached to the tip of the chin, and a lateral X-ray fluoroscopic image was recorded. The sampling frequency was 50 Hz for B4S^™^ and 30 Hz for VF.

For the comparison of the laryngeal movement time captured by the B4S^™^ versus the laryngeal movement time captured by VF, four time points (a–d) were measured for each swallow. On the B4S^™^ waveform, the first timepoint is when Ch2 reaches its first peak value for the first time (a), the second is when Ch1 reaches its first peak value for the first time (b), the third is when Ch1 reaches its second peak value (c), and the fourth is when Ch2 reaches its second peak value (d). On the VF images, the first timepoint is when the larynx is elevated just before the first passage through Ch2 (a′), the second is just before the first passage through Ch1 (b′), the third is just after the second passage through Ch1 (c′), and the fourth is just after the second passage through Ch2 (d′). Next, the durations between a-b, b-c, and c-d measured by the B4S^™^ and those between a’-b’, b’-c’, and c’-d’ on the VF image were calculated. Then, each pair (e.g., between a-b and a’-b’) were compared. The larynx was defined as the upper surface of the airway as the most visualizable area on the VF image. For the VF measurements, two Speech Therapists (KA and YI) evaluated each swallow and then reached a decision by consensus.

Relative and absolute reliability were examined between VF and B4S^™^ times. Relative reliability was examined using the intraclass correlation coefficient (ICC (1.1)). The strength of agreement was interpreted as follows: <0.00, slight; 0–0.19, low; 0.20–0.39, moderate; 0.41–0.69, high; 0.70–0.89, substantial; and 0.90–1.00, very high (Guilford, 1942). Absolute reliability was examined using Bland–Altman analysis (Bland and Altman, 1986) to check the systematic bias and estimate the limit of agreement (LOA). The fixed bias was statistically evaluated using the 95% confidence interval (CI) of the mean differences between the VF time and B4S^™^ time values ( $\bar{d}$ ). A fixed bias was present if zero was not within the range of the 95% CI of $\bar{d}$ . The Bland–Altman plot, in which the distribution of d is biased toward positive or negative, can be used to depict the fixed bias. A proportional bias was present when the value of the VF time minus the B4S^™^ time (d or |d|) was significantly correlated with the mean of the VF time to B4S^™^ time. The magnitude of d or |d| changes depending on the magnitude of the mean of the VF time to B4S^™^ time in the Bland–Altman plot when a proportional bias is present. The 95% LOA was calculated as the mean ±1.96 SD of the differences, and the %LOA was calculated as the mean %d ± 1.96 SD of %d, where %d = (d/mean of the VF time and B4S^™^ time) × 100, using the relative differences between the VF time and B4S^™^ time. These values are shown as Bland–Altman plots. In addition, the 95% CIs of the upper and lower LOAs were calculated.

## Results

**FIGURE 3:**
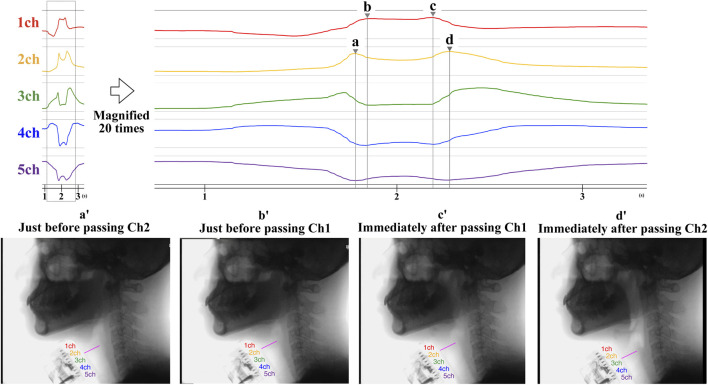
The time measurement method of laryngeal movement captured by B4S™ and VF. The upper figure shows an example of the measured waveform of B4S™. The lower figure shows the corresponding VF image. On the B4S™ waveform, the first time is when Ch2 reaches its first peak value (a), the second is when Ch1 reaches its first peak value (b), the third is when Ch1 reaches its second peak value (c), and the fourth is when Ch2 reaches its second peak value (d). On the VF images, the first time is when the larynx was elevated and immediately before the first passage through Ch2 (a’), the second is when immediately before the first passage through Ch1 (b’), the third is immediately after the time re-passage through Ch1 (c’), and the fourth is when immediately after the time re-passage through Ch2 (d’).

**FIGURE 4:**
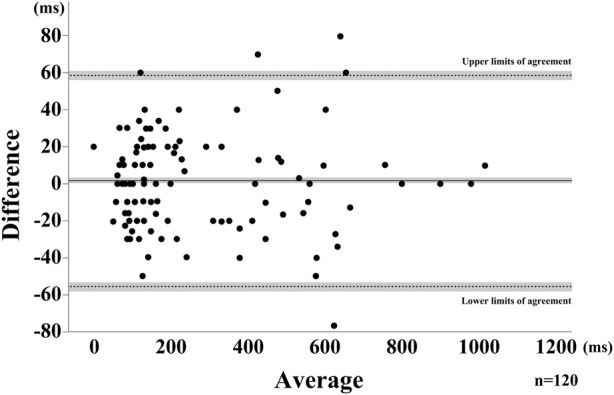
Bland-Altman Plot for the saliva swallow. The horizontal solid line represents the mean, dotted lines represent the LOAs. The shaded areas represent the 95% confidence intervals for the mean and LOAs.

**FIGURE 5:**
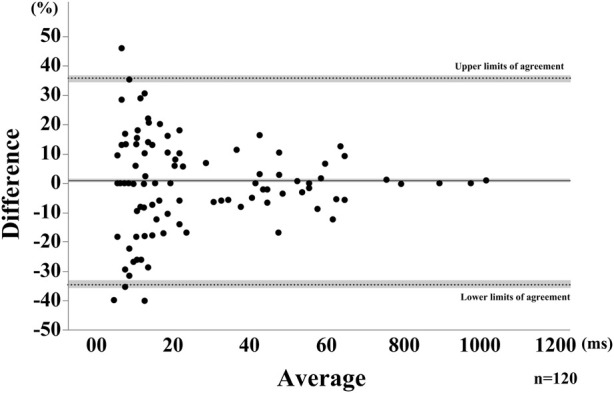
Bland-Altman Plot for the saliva swallow using the relative difference. The horizontal solid line represents the mean, dotted lines represent the LOAs. The shaded areas represent the 95% confidence intervals for the mean and LOAs.

Swallows for all participants were recorded and analyzed for each attempt, for a total of 40 SS. Representative results are shown in Figure 3. B4S^™^ time was 90 ms for a-b, 400 ms for b-c, and 70 ms for c-d, while the VF time was 80 ms for a’-b’, 340 ms for b’-c’, and 110 ms for c’-d’ (Figure 4). The reliability of VF and B4S^™^ times was very high [ICC (1.1) = 0.980]. The Bland–Altman plots are shown in Figures 4, 5, and the data are presented in Tables 1, 2. The 95% CI for the mean of VF time and B4S^™^ time ranged from 0.00 to 3.01, with a fixed error detected in the positive direction. The lower LOA was −55.51 and the 95% CI ranged from −58.12 to −52.89, while the upper LOA was 58.52 and the 95% CI ranged from 55.91 to 61.13. The VF and B4S^™^ time measurements were highly consistent (Table 1; Figure 4). The correlation coefficients for d vs. mean and for |d| vs. mean were r = 0.144 and 0.011, respectively, indicating no proportional error.

**TABLE 1:** Absolute reliability of the SS with Bland-Altman analysis.

	Fixed bias						Proportional bias			
	$\bar{d}$	95%CI	Lower LOA	95%CI	Upper LOA	95%CI	d vs. mean		|d| vs. mean	
							r	*p*	r	*p*
SS	1.50	0.00, 3.01	−55.51	−58.12, −52.89	58.52	55.91, 61.13	0.144	0.116	0.011	0.902

**TABLE 2:** Absolute reliability of the SS with Bland-Altman analysis using the percentage of difference.

Fixed bias						
	%Mean difference	95%CI	Lower %LOA	95%CI	Upper %LOA	95%CI
SS	0.63	0.00, 1.30	−34.46	−33.35, −35.56	35.74	34.63, 36.84

## Discussion

In this study, we examined whether the developed B4S^™^ could capture upward and downward laryngeal movement and accurately display it as waveforms. To that end, we analyzed the agreement between the duration of laryngeal elevation and descent (VF time) and the peak duration of the B4S^™^ waveform (B4S^™^ time) using the ICC and Bland–Altman plots. Quite high ICC was detected in this study, suggesting a strong relative reliability. The 95% CI of the means of B4S^™^ time and VF time ranged from 0.00 to 3.01 in the Bland–Altman showed strong absolute reliability. Those results suggested that the B4S^™^ can capture laryngeal motion with good accuracy.

Although no proportional error was not detected, a fixed error was detected in the positive direction in the Bland-Altman plots. This positive fixed error is considered to be attributed to the difference in sampling frequency between the two instruments used in this study—30 Hz and 50 Hz for VF and B4S^™^, respectively—and thus the time measured by VF is expected to have a larger error from the true value compared with that measured by B4S^™^, which is also one of the reasons for the constant measurement error. Another possible factor affecting positive fixed error may be the measurement method. We used the upper surface of the airway as a landmark to measure laryngeal movement with VF, as it can be more easily and steady visualized than the laryngeal prominence. However, this location is considered slightly different from the laryngeal prominence captured by B4S^™^. In spite of this positive fixed error, the difference between the two measurements was within 10% of the average value, suggesting that the B4S^™^ can accurately detect the duration of superior-inferior laryngeal motion during swallowing, same as previously reported non-invasive device using sEMG, reflective photo sensors, Piezo pressure sensor.

The advantage of B4S^™^ is that it is ready for clinical use in detection of hyolaryngeal dynamics during swallowing as screening and bedside evaluation. The small device and its dedicated tablet is portable and can be feasible at anywhere. The simplicity of simply placing the device on the front neck is almost as easy as four-finger palpation, widely used in clinical dysphagia evaluation. However, intra- and inter-rater reliability issues have been reported for the assessment of laryngeal elevation by palpation (McCullough et al., 2000; Rangarathnam and McCullough, 2016). A previous study reported that patients showing obvious signs of aspiration or penetration were more likely to be evaluated as having less hyolaryngeal elevation, suggesting that the clinician’s impression of hyolaryngeal movement was affected by the patient’s signs of dysphagia (Brates et al., 2020). Intra- and inter-reliability can also be influenced by differences in swallowing performance across swallows. However, it is difficult to confirm such differences by subjective assessment. As supero-inferior laryngeal movements during swallowing can be detected and identified in waveforms as demonstrated with the B4S^™^, the objectivity of evaluation may be ensured, the variability in evaluation caused by differences between the swallows may be resolved, and the influence of clinicians’ impressions on evaluation may be minimized.

The finding that the B4S^™^ accurately depicts laryngeal elevation and descent in waveforms further suggests its potential for providing feedback on swallowing. In dysphagia rehabilitation, providing feedback is considered to enhance the exercise completion and to achieve the techniques for safe and efficient swallow. The effectiveness of biofeedback in enhancing hyoid and laryngeal elevation during swallowing has also been demonstrated (Benfield et al., 2019). In this study, B4S^™^ showed the capability to accurately depict the onset and duration of laryngeal movement during swallowing as a temporal waveform, which is expected to be effective for providing visual feedback for voluntary adjustment of the hyoid and larynx in the real time.

The limitation of this study is that agreement between the B4S^™^ waveforms and actual laryngeal movements were examined only in terms of timing, without considering the distance of motion. The five sensors, which are 5 mm wide, are aligned vertically and spaced approximately 3 mm apart. Although the B4S^™^ may be able to estimate the approximate range of laryngeal elevation from the distance between the most stretched channel at rest and the most stretched channel during swallowing, there is currently no algorithm to measure the exact distance of the movement range. Further study is needed to develop a method for measuring the distance of laryngeal elevation. It is also necessary to investigate the usefulness of this device for evaluation and feedback in clinical settings.

## Conclusion

The B4S^™^ can accurately detect the movement of the larynx during saliva swallow and display it as waveforms. It shows promise as an objective tool for evaluating the larynx during swallowing in medical examinations and clinical evaluations as well as for providing visual feedback of laryngeal movement in swallowing rehabilitation.

## Data Availability

The raw data supporting the conclusion of this article will be made available by the authors, without undue reservation.
